# Quiescence, Stemness and Adipogenic Differentiation Capacity in Human DLK1^−^/CD34^+^/CD24^+^ Adipose Stem/Progenitor Cells

**DOI:** 10.3390/cells10020214

**Published:** 2021-01-22

**Authors:** Florian M. Hatzmann, Asim Ejaz, G. Jan Wiegers, Markus Mandl, Camille Brucker, Stefan Lechner, Tina Rauchenwald, Marit Zwierzina, Saphira Baumgarten, Sonja Wagner, Monika Mattesich, Petra Waldegger, Gerhard Pierer, Werner Zwerschke

**Affiliations:** 1 Division of Cell Metabolism and Differentiation Research, Research Institute for Biomedical Aging Research, University of Innsbruck, Rennweg 10, A-6020 Innsbruck, Austria; florian.hatzmann@uibk.ac.at (F.M.H.); ejaza@upmc.edu (A.E.); markus.mandl@uibk.ac.at (M.M.); Camille.Brucker@uibk.ac.at (C.B.); Stefan.Lechner@medel.com (S.L.); s.f.baumgarten@medisin.uio.no (S.B.); Sonja.Wagner@i-med.ac.at (S.W.); Petra.Waldegger@uibk.ac.at (P.W.); 2 Center for Molecular Biosciences Innsbruck, University of Innsbruck, Innrain 80-82, A-6020 Innsbruck, Austria; 3 Department of Plastic Surgery, University of Pittsburgh Medical Center, 3550 Terrace Street, 6B Scaife Hall, Pittsburgh, PA 15261, USA; 4 Division of Developmental Immunology, Biocenter, Medical University of Innsbruck, Innrain 80-82, A-6020 Innsbruck, Austria; jan.wiegers@i-med.ac.at; 5 Department of Plastic, Reconstructive and Aesthetic Surgery, Medical University of Innsbruck, Anichstraße 35, A-6020 Innsbruck, Austria; tina.rauchenwald@tirol-kliniken.at (T.R.); marit.zwierzina@tirol-kliniken.at (M.Z.); mattesich@dpch.at (M.M.); gerhard.pierer@tirol-kliniken.at (G.P.)

**Keywords:** ex vivo, human adipose stem/progenitor cells, quiescence, senescence, stemness, proliferation, adipogenesis

## Abstract

We explore the status of quiescence, stemness and adipogenic differentiation capacity in adipose stem/progenitor cells (ASCs) ex vivo, immediately after isolation from human subcutaneous white adipose tissue, by sorting the stromal vascular fraction into cell-surface DLK1^+^/CD34^−^, DLK1^+^/CD34^dim^ and DLK1^−^/CD34^+^ cells. We demonstrate that DLK1^−^/CD34^+^ cells, the only population exhibiting proliferative and adipogenic capacity, express ex vivo the bonafide quiescence markers p21^Cip1^, p27^Kip1^ and p57^Kip2^ but neither proliferation markers nor the senescence marker p16^Ink4a^. The pluripotency markers NANOG, SOX2 and OCT4 are barely detectable in ex vivo ASCs while the somatic stemness factors, c-MYC and KLF4 and the early adipogenic factor C/EBPβ are highly expressed. Further sorting of ASCs into DLK1^−^/CD34^+^/CD24^−^ and DLK1^−^/CD34^+^/CD24^+^ fractions shows that KLF4 and c-MYC are higher expressed in DLK1^−^/CD34^+^/CD24^+^ cells correlating with higher colony formation capacity and considerably lower adipogenic activity. Proliferation capacity is similar in both populations. Next, we show that ASCs routinely isolated by plastic-adherence are DLK1^−^/CD34^+^/CD24^+^. Intriguingly, CD24 knock-down in these cells reduces proliferation and adipogenesis. In conclusion, DLK1^−^/CD34^+^ ASCs in human sWAT exist in a quiescent state, express high levels of somatic stemness factors and the early adipogenic transcription factor C/EBPβ but senescence and pluripotency markers are barely detectable. Moreover, our data indicate that CD24 is necessary for adequate ASC proliferation and adipogenesis and that stemness is higher and adipogenic capacity lower in DLK1^−^/CD34^+^/CD24^+^ relative to DLK1^−^/CD34^+^/CD24^−^ subpopulations.

## 1. Introduction

Adipose stem/progenitor cells (ASCs) play a crucial role in adipose tissue homeostasis, regeneration [1,2,3,4], expansion [5] and aging [6,7,8,9]. Beside adipocyte hypertrophy/hypotrophy, proliferation and terminal adipogenic differentiation of ASCs are the main causes for the high degree of plasticity in white adipose tissue (WAT), which is unique among organs in adults and crucial for the maintenance of metabolic health [10]. Furthermore, over the past two decades, WAT has emerged as an alternative tissue source for stromal cells in translational regenerative medicine and regarding the multipotent differentiation potential and regenerative properties of adult stem cells [11,12,13,14]. The mechanisms regulating ASC self-renewal and differentiation are a current focus of research into obesity, aging and regenerative medicine.

In subcutaneous (s)WAT, ASCs reside at the vascular interface surrounding small blood vessels [15,16,17]. These cells can be isolated from the stromal vascular fraction (SVF). Sorting of SVF cells for surface staining of the stem cell markers Delta-like protein 1 (DLK1)/Preadipocyte factor-1 (Pref-1) [18,19] and Cluster of differentiation 34 (CD34) [20,21] identifies three main subpopulations: DLK1^+^/CD34^−^, DLK1^+^/CD34^dim^ and DLK1^−^/CD34^+^ cells [16]. Only the latter shows a DLK1^−^/CD34^+^/CD90^+^/CD45^−^/CD31^−^ immune phenotype and exhibits proliferative and adipogenic differentiation capacity [16]. This ASC population still contains heterogeneous cell types and its further subclassification is important. Another interesting ASC surface marker, which was used to characterize murine ASCs, is CD24 [2], a glycosylphosphatidylinositol-linked cell surface receptor [22]. CD24 is expressed by several stem cell types, plays a role in the regulation of cell proliferation and differentiation [23,24] and tracks various pluripotent states in mouse and human cells [25]. The functional importance of CD24 in human ASCs remains to be elucidated.

Quiescence, a state of reversible proliferation arrest, is a common feature of adult stem cells that increases stress resistance and most likely self-renewal capacity [21,22]. Although the turnover of adipocytes in sWAT of adult humans is low [1,9], suggesting that ASCs proliferate at a low rate and presumably reside predominantly in a quiescent state in sWAT; this has so far not been demonstrated. In general, quiescent cells leave the cell cycle and undergo a reversible G0-phase arrest preventing proliferation and differentiation. Studies in hematopoietic, muscle and neural stem cells showed that the cyclin-dependent kinase inhibitors (CDKIs) p21^Cip1^, p27^Kip1^ and p57^Kip2^ play a key role in inducing and maintaining quiescence [26,27]. Beside the presence of CDKIs, the absence of differentiation, senescence (e.g., p16^Ink4a^) and proliferation markers (e.g., Ki67, cyclins) characterize the quiescent state.

Features linked to quiescence in stem cells are a high self-renewal capacity and tissue regeneration potential. The core regulatory network suppressing differentiation and maintaining pluripotency of embryonic stem cells consists of the three transcription factors NANOG, SOX2 and OCT4 [28]. Contradictory results exist regarding the expression and importance of these factors in adult stem cells including ASCs [29,30,31,32,33,34,35]; however, their expression has so far not been analyzed ex vivo in human ASCs immediately after their isolation from sWAT but only in in vitro expanded passages of ASCs. Two additional transcription factors, c-MYC and KLF4, belonging to the four somatic cell reprogramming factors together with OCT4 and SOX2 [36,37], have been shown to ensure high stemness in hematopoietic stem cells by balancing quiescence/proliferation (self-renewal) and differentiation [38,39]. c-MYC is crucial for inhibiting terminal differentiation [38,40] and KLF4 inhibits cell cycle progression and is involved in the proliferation/differentiation switch [41,42,43,44]. Both transcription factors are transiently induced in murine 3T3-L1 preadipocytes as very early genes after induction of adipogenesis [45,46]. It was suggested that the expression of c-MYC is a hub at which growth arrested 3T3-L1 cells are either directed toward cell cycle entry or committed to terminal differentiation [47]. KLF4 was identified as a negative regulator of proliferation and an activator of adipogenesis in 3T3-L1 cells, inducing the early pro-adipogenic regulator CCAAT/enhancer-binding protein β (C/EBPβ) [46]. Thereupon, C/EBPβ induces the adipogenic key factor peroxisome proliferator-activated receptor-γ2 (PPARγ2) and additional members of the C/EBP family [48] leading to activation of the adipogenic program to acquire the adipocyte phenotype [49]. The role of c-MYC and KLF4 in human ASCs is unknown and the expression of early transcription factors regulating transition from cell cycle arrest to terminal differentiation, such as C/EBPβ, are little studied ex vivo in ASCs immediately after their isolation from human sWAT.

The aims of the present study are to analyze the importance of CD24 and the status of quiescence, stemness, proliferation and adipogenic differentiation capacity in ASCs ex vivo, immediately after their isolation from human sWAT.

## 2. Materials and Methods

### 2.1. Donors

Human sWAT samples were taken from patients undergoing abdominoplasty at the Department of Plastic, Reconstructive and Aesthetic Surgery at the Medical University of Innsbruck, Austria. All patients gave their written informed consent and the study has been approved by the Ethics Committee of Medical University of Innsbruck, Austria, according to the Declaration of Helsinki (ethic committee board number: AN 3047 257/4.13). All sWAT samples were obtained from the lower abdomen (low transverse incision) from the subcutaneous fat layer between the rectus sheath and the fascia of scarpa. None of the patients had malignant or severe metabolic diseases or were under any hormone therapy. In total ASCs isolated from sWAT of 16 donors (12 females, 4 males) were used for the experiments. Clinical anthropometric parameters of the donors can be found in Supplementary Table S1.

### 2.2. Isolation of the Stromal Vascular Fraction (SVF) from Subcutaneous White Adipose Tissue

Abdominal sWAT samples were processed in a laminar flow sterile working bench class II. After washing the tissue samples in PBS, blood vessels and connective fibrous material were dissected and the tissue was cut into small pieces (⌀ ~ 5 mm) to be 90 min incubated at 37 °C in digestion buffer consisting of 200 U/mL collagenase (CLS Type I, Worthington Biochemical Corp., Lakewood, NJ, USA) and 2% *w/v* BSA in PBS. The dispersed cells were poured through a sieve to remove remaining connective tissue and centrifuged at 200× *g* for 10 min. The pelleted cells were resuspended in erythrocyte lysis buffer (0.155M Na_4_Cl, 5.7 Mm K_2_HPO_4_, 0.1 mM EDTA, pH 7.3), incubated at room temperature for 10 min and filtered through 100 µm cell strainer. After centrifugation, the resuspended cells were filtered through 35 µm cell strainer to obtain pure single cell suspensions. The resulting cell population is referred to as the SVF and was suspended in DMEM/F-12 medium with HEPES and l-glutamine (Gibco, Vienna, Austria) containing 33 µM biotin, 17 µM pantothenate and 20 µg/mL Ciprofloxacin) supplemented with 10% FBS (Sigma, Vienna, Austria) which we refer to as ASC2 medium. For standard isolation of ASCs by plastic-adherence, SVF was seeded in ASC2 in 6-well plates at a density of 70,000 cells/cm^2^. After 16 h, medium was changed to serum-free ASC medium for 6 days and non-adherent cells were continuously removed through washing. The remaining cells are referred to as passage -1 (P-1) and were re-seeded in ASC2 medium. After 16 h, medium was changed to proliferation medium PM4 (DMEM/F-12 medium with HEPES and l-glutamine containing 33 µM biotin, 17 µM pantothenate, 10 ng/mL EGF, 1 ng/mL FGF, 500 ng/mL Insulin and 20 µg/mL Ciprofloxacin, supplemented with 2.5% FBS) for amplification. At 70% confluence these cells were harvested, now referred to as P0, frozen and stored in liquid nitrogen. Re-thawed cell aliquots taken into culture were referred to as P1.

### 2.3. Flow Cytometric Sorting and Analyses of Freshly Isolated Cells

The SVF was isolated as described above and cells were directly subjected to immunofluorescence staining with anti-CD34-PE-Cy7 (BD Pharmingen, #556626), a rat monoclonal anti-human-DLK1/PREF1 antibody (Adipogen, AG-25A-0091) along with an anti-rat-APC antibody (BD Pharmingen, #551019) and anti-CD24-BV421 (Biolegend, #311121). Cells were sorted using a FACS Aria machine (BD, Bioscience, Vienna, Austria) and collected in ASC2 medium. Directly after sorting, cells were counted and immediately used for experiments.

### 2.4. Flow Cytometric Analysis of Cultivated ASCs

ASCs were processed for several passages in cell culture. For FACS analysis, cells were trypsinized and washed with PBS before immunofluorescence staining with anti-CD24-BV421 (Biolegend, #311121). For intracellular staining, cells were permeabilized using the BD Cytofix/Cytoperm Kit (BD Bioscience, #554714, Vienna, Austria). Cells were measured using a FACS Canto II (BD Bioscience, Vienna, Austria) and analyzed using FlowJo 10.5.2 software.

### 2.5. Cell Culture

Cells were always seeded in ASC2 medium and after 16 h the medium was changed either to proliferation medium PM4 (DMEM/F-12 medium with HEPES and l-glutamine containing 33 µM biotin, 17 µM pantothenate, 10 ng/mL EGF, 1 ng/mL FGF, 500 ng/mL Insulin and 20 µg/mL Ciprofloxacin) supplemented with 2.5% FBS or to serum-free medium ASC1 (DMEM/F-12 medium with HEPES and l-glutamine containing 33 µM biotin, 17 µM pantothenate and 20 µg/mL Ciprofloxacin) depending on the experimental setup. 

### 2.6. Adipogenic Differentiation

For adipogenic differentiation, cells were seeded densely and after cell adherence the medium was changed to ASC1 for 48 h in order to synchronize the cells. In confluent cells adipogenic differentiation was induced by adding differentiation medium (ASC1 containing 0.2 µM Insulin, 0.25 µM Dexamethasone, 10 µg/mL Transferrin, 0.5 mM IBMX and 2.5% FBS). After 3 days and 6 days the medium was changed to differentiation medium without IBMX. Subsequently, cells were cultivated until d9 when RNA or protein samples were taken. Oil Red-O staining was performed to visualize lipid droplets that are formed in differentiated adipocytes. For Oil Red-O staining, cells were seeded in standing drops with 15,000 cells per drop. On day 9 or day 14 post induction of adipogenesis cells were fixed in 4% paraformaldehyde in PBS for 1 h, washed with PBS and stained with 0.5% Oil Red-O in Isopropanol/water (60:40) for 1 h at room temperature. After final washing steps with aqua dest representative pictures of the stained cells were taken and staining was quantified using ImageJ 1.51n software.

### 2.7. Real-Time Quantitative PCR

RNA was isolated using the RNeasy Plus Micro Kit (Qiagen, #74034, Hilden, Germany) according to the manufacturer´s instructions and RNA concentration was measured by Nano Drop (Thermo Scientific, Vienna, Austria). The First Strand cDNA Synthesis Kit (Thermo, #1622, Vienna, Austria) was used for reverse cDNA synthesis according to the manufacturer´s protocol. For cDNA synthesis 200ng RNA were used and cDNA was diluted 1:5 for real-time quantitative PCR (RT-qPCR). Gene expression was measured with a QuantStudio^TM^ 7 Flex System (Thermo Fisher Scientific, Vienna, Austria) using AceQ SYBR Green Master Mix (Vazyme, Q111-02, Nanjing, China). The number of biological replicates (i.e., donors) per group was n = 3 for every experiment and all samples were measured in technical triplicates. All primer sequences can be found in Supplementary Table S2. Gene expression was calculated as e = 2^-Δct and is shown as relative gene expression normalized to the highest expressed gene which was set to 100.

### 2.8. Western Blot Analysis

For protein analysis, cells were harvested in SDS lysis buffer and sonicated. The determination of protein concentration was done using the Compat-Able Protein Assay Preparation Reagent Set (Thermo Scientific, #23215, Vienna, Austria) and the Pierce BCA Protein Assay Kit (Thermo Scientific, #23227, Vienna, Austria). Samples (20 µg total protein) were separated on a 10% SDS-polyacrylamide gel and blotted onto a PVDF membrane. The membranes were probed with anti-perilipin antibody (Cell Signaling, #9349, Frankfurt am Main, Germany), anti-PPARγ2 antibody (Cell Signaling, #2435, Frankfurt am Main, Germany), anti-FABP4 antibody (Cayman, #10004944, Tallinn, Estonia), anti-CD24 antibody (Abcam, #179821) and anti-GAPDH (Thermo Scientific, AM4300, Vienna, Austria). Goat anti-rabbit IgG-HRP (DAKO, Vienna, Austria) and goat anti-mouse IgG-HRP (Promega, Walldorf, Germany) served as secondary antibodies and signals were detected with a chemiluminescence detection system. Membranes were stained with Ponceau S for normalization to total protein. Densitometric analysis was done using ImageJ software.

### 2.9. Proliferation Assay

After sorting, cells were seeded in 6-well plates with a density of 30,000 cells per well or in 48-well plates with a density of 5000 cells per well in ASC medium containing 10% FBS. After 16 h, medium was changed to PM4. Medium was changed every 2–3 days and cells were harvested for counting on indicated time points. Therefore, medium from the wells was removed, cells were washed once with PBS and 500 µL of trypsin were added to each well. The cells were incubated with trypsin at 37 °C for 5 min or until cells were detached. Then 1 mL of ASC medium containing 10% FBS was added and the 1.5 mL in each well were transferred into FACS tubes. The relative cell number was measured by acquiring the number of events recorded in 90 seconds at a medium flow rate using a FACS Canto II (BD, Bioscience, Vienna, Austria) or cells were counted using a Neubauer chamber.

### 2.10. BrdU Assay

5-Bromo-2´-deoxy-uridine Labeling and Detection Kit III (Roche, #11444611001, Vienna, Austria) was used and the assay was performed as described in the manufacturer´s manual. Briefly, cells were seeded in 96-well plates with a density of 1600 cells per well in ASC2 medium. After 16h, medium was changed to PM4 supplemented with BrdU (final concentration 10 µM). Control wells without BrdU were included. After 6 days of incubation the cells were fixed with 70% ethanol + 0.5M HCl at −20 °C for 30 min. In the following, cells were incubated with Nuclease working solution at 37 °C without CO_2_ for 30 min. Between the steps cells were washed with PBS+10% FBS several times. Then, cells were stained with anti-BrdU-POD Fab fragments diluted in washing buffer + 10 mg/mL BSA (final concentration anti-BrdU-POD 200 mU/mL) at 37 °C without CO_2_ for 30 min. POD substrate was added and after 30 min of incubation at room temperature the absorption at 490 nm was measured.

### 2.11. Cell Cycle Analysis

Propidium Iodide (PI) staining was used to analyze cell cycle. ASCs in passage 3 were seeded in a density of 100,000 cells per 6-well in ASC2 medium. After 16 h, medium was changed to PM4 and on day 4 post seeding cells were harvested for analysis. After trypsinizing and centrifugation, cells were resuspended in fixation solution (70% ethanol in PBS), again centrifuged and then resuspended in PI-solution (0.1% Triton X-100, 0.1% sodium citrate, 50 µg/mL propidium iodide in Aqua bidest). After 30 min of incubation in the dark at room temperature, cells were analyzed by using a FACS Canto II (BD, Bioscience, Vienna, Austria).

### 2.12. Colony Formation Assay

To test the self-renewal capacity of CD34^+^/DLK1^−^ ASCs, 1000 cells per well were seeded directly after sorting in 6-well plates in ASC2 medium. After 16h, when cells were attached, medium was changed to PM4. Medium was changed every 2–3 days. On day 15 post seeding the cells were fixed with methanol/acetone (1:1) for 10 min and stained with crystal violet for 15 min to count colonies.

### 2.13. shRNA Mediated CD24 Knock Down

To knock down CD24, pLKO.1 vectors encoding shRNAs targeting the human CD24 gene were purchased from a commercial supplier (Dharmacon, Lafayette, LA, USA). sh1 (TRCN0000057520), sh2 (TRCN0000057522) and a non-targeting control (scr) were used. All plasmids were amplified in *E. coli* Stbl3 and endotoxin-free plasmids were prepared using the Endo Free Plasmid Maxi Kit (Qiagen, #12362, Hilden, Germany). Generation of lentiviral particles and infection of ASCs was performed as described previously [50].

### 2.14. Statistics

All experiments were replicated with at least three donors. For statistical analysis, unpaired student´s *t*-test was performed. Significances are indicated like this: * = *p* < 0.05; ** = *p* < 0.01; *** = *p* < 0.001

## 3. Results

### 3.1. Isolation of ASC Populations Defined by DLK1, CD34 and CD24 from Human sWAT

**Figure 1:**
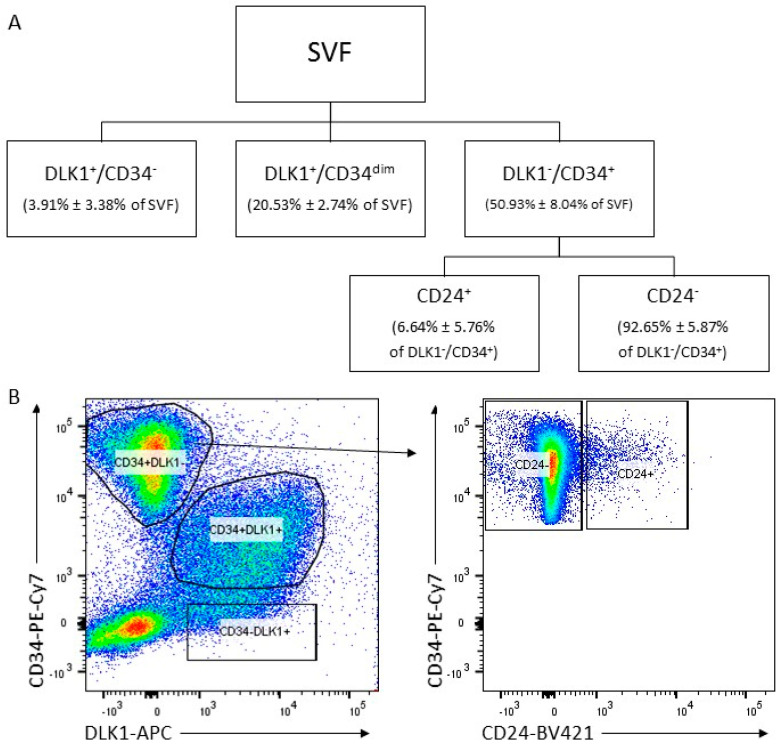
Isolation of DLK1^−^/CD34^+^/CD24^+^ and DLK1^−^/CD34^+^/CD24^−^ cell populations from the SVF of human sWAT. (**A**) Fluorescence-activated cell sorting (FACS) scheme: Un-permeabilized and un-fixed cells in the SVF were sorted for cell surface tethered DLK1 and CD34 protein by staining with anti-DLK1 and anti-CD34 antibodies to obtain DLK1^+^/CD34^−^ (3.91% ± 3.38%), DLK1^+^/CD34^dim^ (20.53% ± 2.74%) and DLK1^−^/CD34^+^ (50.93% ± 8.04%) cells. These cells were subsequently sorted for cell surface tethered CD24 generating the DLK1^−^/CD34^+^/CD24^+^ (6.64% ± 5.76%) and DLK1^−^/CD34^+^/CD24^−^ (92.65% ± 5.87%) fractions. n = 4. (**B**) Representative Dot Blots showing the sorting scheme to obtain DLK1^−^/CD34^+^/CD24^+^ and DLK1^−^/CD34^+^/CD24^−^ cell populations.

To better characterize ASCs in human sWAT, we analyzed cell populations defined by the cell surface proteins DLK1, CD34 and CD24 ex vivo. To do this, the SVF was generated from fresh sWAT and immediately subjected to fluorescence-activated cell sorting for DLK1^+^/CD34^−^, DLK1^+^/CD34^dim^ and DLK1^−^/CD34^+^ cell surface populations (Figure 1A). Only the DLK1^−^/CD34^+^ cells, which constitute ~80% of the three fractions and ~55% of all SVF cells, express the ASC marker profile DLK1^−^/CD34^+^/CD90^+^/CD45^−^/CD31^−^ and possess colony formation activity and high proliferative and adipogenic differentiation capacity [16]. This fraction was further sorted into cell surface CD24^+^ and CD24^−^ populations. Approximately 94.5% of the DLK1^−^/CD34^+^ cells are CD24^−^ and 5.5% CD24^+^ (Figure 1B). This suggests that ~3.0% of all SVF cells are CD24^+^. Samples from several different donors (Supplementary Table S1) were subjected to functional analyses directly after sorting.

### 3.2. DLK^−^/CD34^+^ ASCs in Human sWAT Are in a Quiescent State and Express High Levels of the Stemness Factors KLF4 and c-MYC and of the Early Pro-Adipogenic Factor C/EBPβ

**Figure 2:**
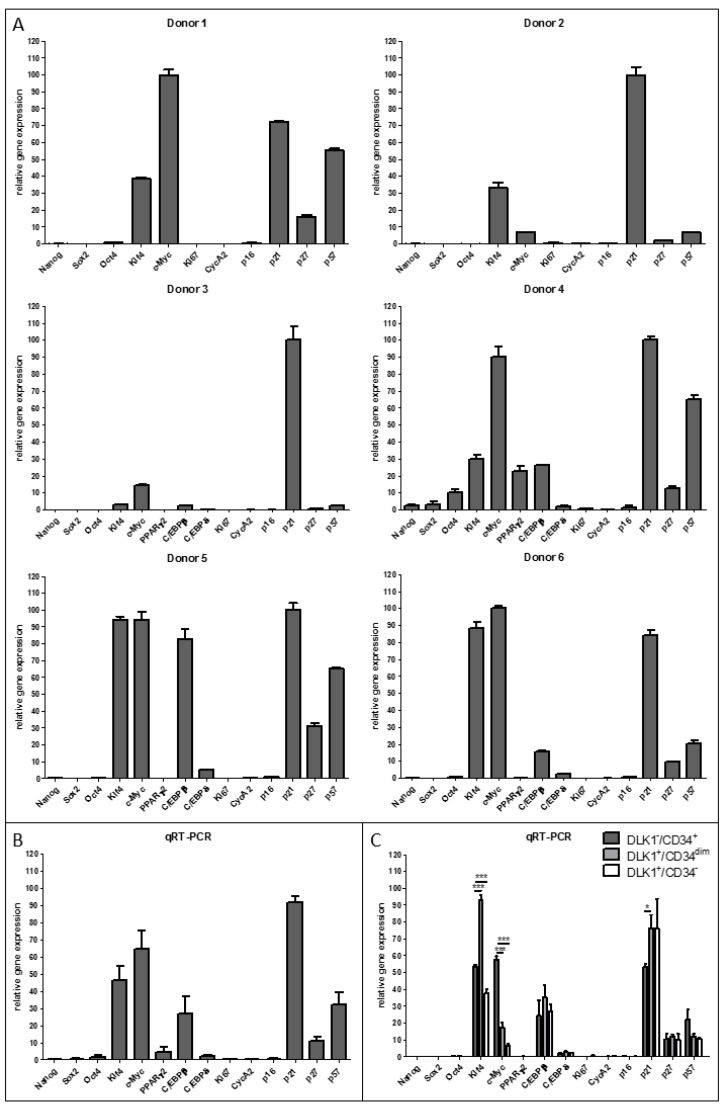
Analysis of the expression of markers for quiescence, proliferation, senescence, stemness and adipogenic differentiation in DLK1^−^/CD34^+^ ASCs. Cells were lyzed directly after sorting, RNA was isolated and RT-qPCR performed to analyze the mRNA expression of Cyclin A2, Ki67 (proliferation), p16^INK4a^ (senescence), p21^Cip1^, p27^Kip1^, p57^Kip2^ (quiescence), NANOG, OCT4, SOX2 (pluripotency), KLF4, c-MYC, (stemness), PPARγ2, C/EBPβ and C/EBPδ (adipogenesis). (**A**) The gene expression in DLK1^−^/CD34^+^ ASCs derived from six different donors is shown. (**B**) The results of the six donors are merged. (**C**) The gene expression in DLK1^−^/CD34^+^, DLK1^+^/CD34^dim^ and DLK1^+^/CD34^−^ cell populations is compared. Gene expression is normalized to RPS18. Mean + SEM is shown, n = 3; unpaired student’s t-test was performed to calculate significances; * *p* < 0.05 and *** *p* < 0.001.

The quiescence state is a reversible proliferation arrest that is important for stress resistance and self-renewal capacity of adult stem cells. To explore whether DLK1^−^/CD34^+^ ASCs in sWAT are in a quiescent state, mRNA was prepared immediately after sorting of ASC populations from 6 different donors and analyzed for the expression of p21^Cip1^, p27^Kip1^ and p57^Kip2^ (Figure 2A,B). The 3 CDKIs were considerably expressed in DLK1^−^/CD34^+^ ASCs of all 6 donors. p21^Cip1^ mRNA levels were always the highest followed by p57^Kip2^ and p27^Kip1^. While the p21^Cip1^ mRNA expression levels were constantly high, the expression level of p57^Kip2^ and p27^Kip1^ fluctuated more, revealing donor dependent variations. ASCs from 3 donors showed similar levels of p21^Cip1^ and p57^Kip2^ mRNA while others contained over 10 times more p21^Cip1^ than p57^Kip2^ mRNA. p27^Kip1^ was always considerably lower expressed than p21^Cip1^. Both the proliferation marker Ki67, which is known to be expressed in all phases of the cell cycle except G0 and the S-phase marker Cyclin A2 were not detectable (Figure 2A). The senescence marker p16^Ink4a^ was either undetectable or expressed at a very low level (Figure 2A). In conclusion, all ASCs in sWAT express considerable levels of p21^Cip1^, p57^Kip2^ and p27^Kip1^ and neither proliferation nor senescence markers (Figure 2B), indicating that they are most likely in a quiescent state.

The pluripotency markers NANOG, SOX2 and OCT4 were barely detectable in ASCs isolated from 5 out of 6 donors. One donor had ASCs showing low but significant expression of NANOG, SOX2 and OCT4 mRNA (Figure 2A,B). We conclude, in the vast majority of donors NANOG, SOX2 and OCT4 are hardly detectable in ASCs of sWAT.

In contrast to the pluripotency markers, the two somatic cell reprogramming factors, c-MYC and KLF4, which have been shown to ensure high stemness in human hematopoietic stem cells (HSCs) [38,44], were strongly expressed in DLK1^−^/CD34^+^ ASCs derived from 6 out of 6 donors (Figure 2A,B). In 3T3-L1 preadipocytes, KLF4 acts as a negative regulator of proliferation and an activator of the early adipogenic transcription factor C/EBPβ [46]. The analysis in DLK1^−^/CD34^+^ ASCs derived from 4 different donors showed that C/EBPβ was always considerably expressed together with c-MYC and KLF4 (Figure 2A,B), suggesting an active KLF4 axis in these cells. The early adipogenic C/EBP family transcription factor C/EBPδ was also detectable in ASCs from 4 out of 4 donors but at much lower levels. The adipogenic key transcription factor peroxisome proliferator-activated receptor-γ2 (PPARγ2) was undetectable in ASCs derived from sWAT of 3 out of 4 donors while ASCs derived from a fourth donor expressed PPARγ2 at the same level as C/EBPβ.

In summary, our data show a scenario, in which proliferation markers are not expressed but the anti-proliferative CDKIs p21^Cip1^, p57^Kip2^ and p27^Kip1^, the pro-proliferative transcription factor c-MYC, the terminal differentiation promoting transcription factors KLF4 and the early pro-adipogenic factor C/EBPβ are expressed in DLK1^−^/CD34^+^ ASCs of human sWAT. This indicates a balance between proliferation and differentiation and eventually the maintenance of the quiescent state in the ASCs while major pluripotency factors are barely expressed. Thus, the vast majority of DLK1^−^/CD34^+^ ASCs in sWAT consists most likely of quiescent cells with high stemness but no pluripotency.

### 3.3. Expression of Quiescence, Proliferation, Senescence, Stemness and Adipogenesis Markers in DLK1^−^/CD34^+^ Relative to DLK1^+^/CD34^dim^ and DLK1^+^/CD34^−^ SVF Cell Populations

The comparison of DLK1^−^/CD34^+^ ASCs with the two other DLK1/CD34 defined subpopulations (DLK1^+^/CD34^−^ and DLK1^+^/CD34^dim^) reveals hardly any differences in the expression pattern of the quiescence, proliferation, senescence, stemness and adipogenic differentiation markers analyzed (Figure 2C). We see only some gradual differences. c-MYC is significantly higher (~70%) and p21^Cip1^ significantly lower (~50%) expressed in DLK1^−^/CD34^+^ ASCs than in DLK1^+^/CD34^−^ and DLK1^+^/CD34^dim^ cells. Moreover, the pro-adipogenic factor KLF4 is significantly higher expressed in the two CD34 positive cell types (DLK1^+^/CD34^dim^ and DLK1^−^/CD34^+^ cells) than in DLK1^+^/CD34^−^ cells. Thus, all DLK1/CD34 defined subpopulations in the SVF of sWAT consist of quiescent cells expressing somatic stemness factors. Despite the similarities, higher c-MYC and lower p21^Cip1^ expression levels in DLK1^−^/CD34^+^ ASCs might reflect their known proliferative capacity in contrast to the other two cell types [16]. Moreover, the higher level of the very early adipogenic factor KLF4 in the CD34^+^ cell types could mirror a better adipogenic differentiation capacity.

### 3.4. Stemness Is Higher and Adipogenic Differentiation Potential Lower in Human DLK^−^/CD34^+^/CD24^+^ than in DLK1^−^/CD34^+^/CD24^−^ ASC Populations

**Figure 3:**
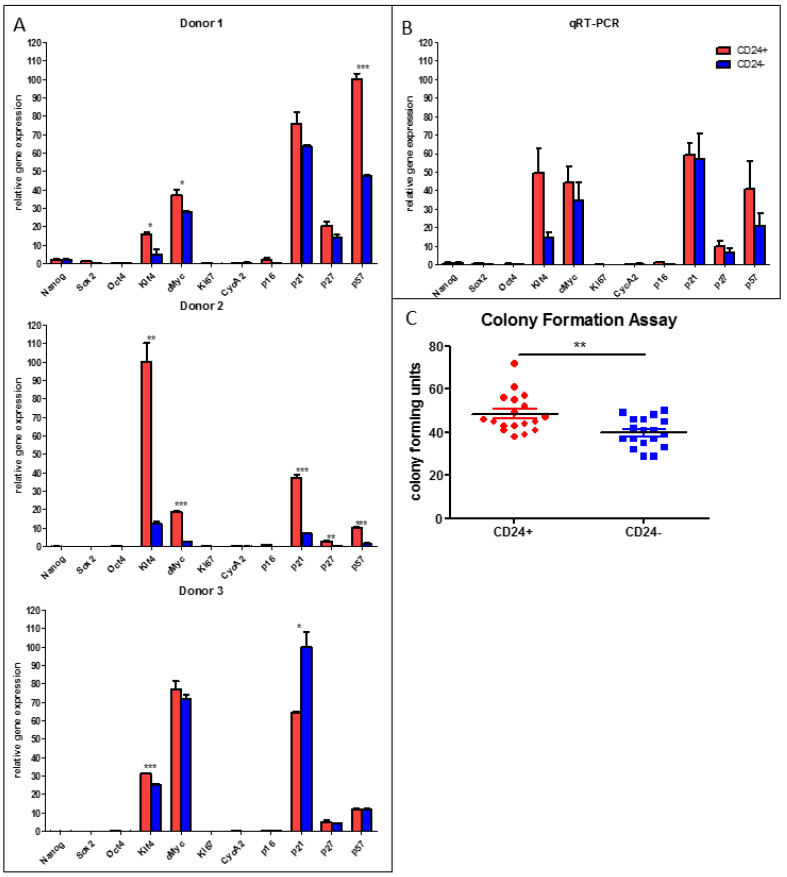
Analysis of the expression of markers for quiescence, proliferation, senescence, stemness and adipogenesis in DLK1^−^/CD34^+^/CD24^+^ and DLK1^−^/CD34^+^/CD24^−^ adipose stem/progenitor cells (ASCs). Cells were lyzed directly after sorting, RNA was isolated and RT-qPCR performed to analyze the mRNA expression of Cyclin A2, Ki67 (proliferation), p16^INK4a^ (senescence), p21^Cip1^, p27^Kip1^, p57^Kip2^ (quiescence), NANOG, OCT4, SOX2 (pluripotency), KLF4 and c-MYC (stemness). (**A**) The gene expression in ASCs derived from three different donors is shown. (**B**) The results of the three donors are merged. Mean + SEM is shown, n = 3, gene expression normalized to RPS18, unpaired student´s t-test was performed to calculate significances. (**C**) Colony Formation Assay in CD24^+^ and CD24^−^ ASCs. 1000 cells per well were seeded in 6-well plates and colonies were counted 15 days post seeding; n = 18 from 3 donors, Mean + SEM is shown; unpaired student´s t-test was performed to calculate significances. * *p* < 0.05, ** *p* < 0.01 and *** *p* < 0.001.

**Figure 4:**
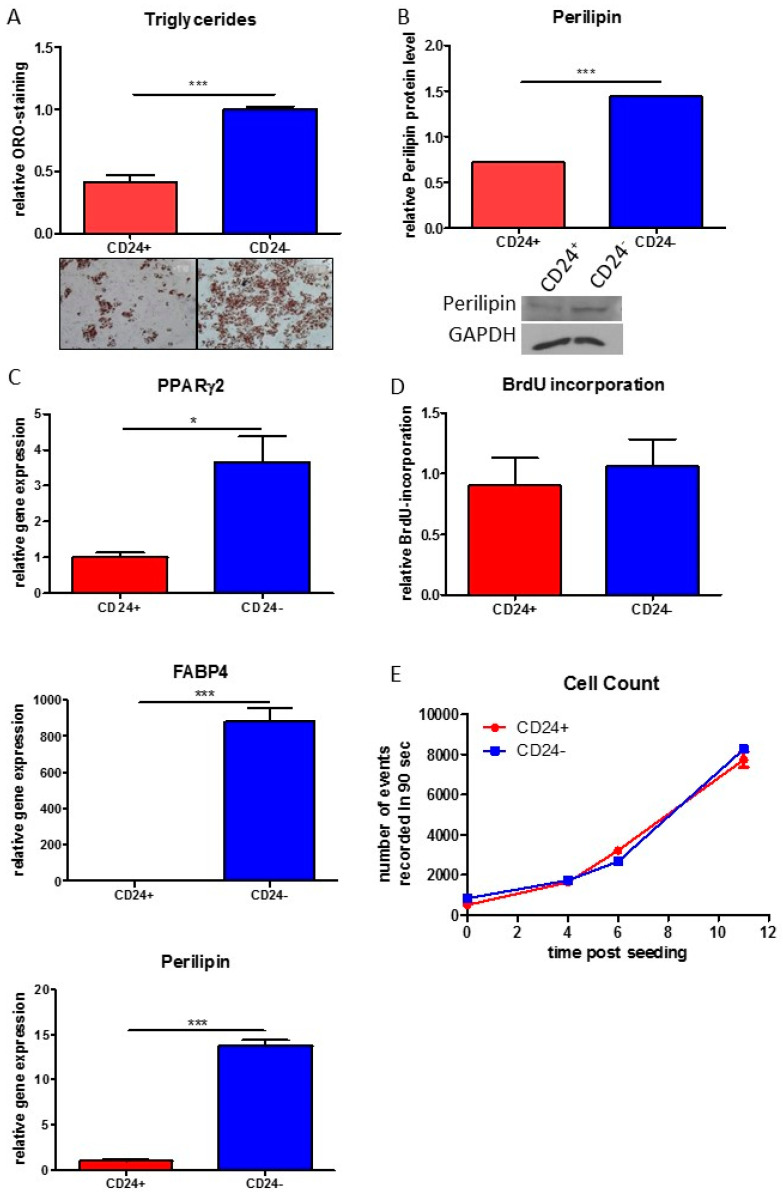
Adipogenic differentiation and proliferation in DLK1^−^/CD34^+^/CD24^+^ and DLK1^−^/CD34^+^/CD24^−^ ASCs. Cells were seeded directly after sorting in 48-well plates and adipogenesis was induced 16 h later. Representative results for ASCs from 3 different donors are shown 9 days post induction of adipogenesis. (**A**) Oil Red-O (ORO) staining. (**B**) Western Blot analysis of perilipin protein level. (**C**) RT-qPCR gene expression analysis of PPARγ2, FABP4 and Perilipin. Gene expression was normalized to RPS18 as control. (**D**) BrdU incorporation assay. 1600 cells/96-well plate were seeded directly after sorting and BrdU incorporation was measured 6 days after adding BrdU. (**E**) Cell counting. 5000 cells/48-well plate were seeded and the relative cell numbers counted by FACS at the indicated time points. Representative results from 3 different donors are shown. Mean+SEM is shown; unpaired student´s t-test was performed to calculate significances; * *p* < 0.05 and *** *p* < 0.001.

While CD24 is considered as a marker for high stem-like capacity of murine adipose stem cells [2], the importance of CD24 in human ASCs is unknown. We compared the expression of quiescence, proliferation, senescence and stemness markers in freshly isolated DLK1^−^/CD34^+^/CD24^+^ versus DLK1^−^/CD34^+^/CD24^−^ ASCs (Figure 3A,B). We found no significant differences between the two ASC types regarding the expression levels of the quiescence markers p21^Cip1^, p27^Kip1^ and p57^Kip2^, the proliferation markers Ki67 and Cyclin A2, the senescence marker p16^Ink4a^ and the pluripotency markers NANOG, SOX2 and OCT4 (Figure 3A). However, both stemness factors, KLF4 and c-MYC, are significantly higher expressed in DLK1^−^/CD34^+^/CD24^+^ ASCs (Figure 3A,B). This correlates with a significant higher colony formation capacity (Figure 3C) and a considerably lower adipogenic differentiation activity of DLK1^−^/CD34^+^/CD24^+^ relative to DLK1^−^/CD34^+^/CD24^−^ ASCs (Figure 4). Oil Red-O staining on day 9 post induction of adipogenesis indicates dramatically higher accumulation of triglycerides in DLK1^−^/CD34^+^/CD24^−^ ASCs (Figure 4A) and Western Blot analysis shows higher levels of the adipocyte marker perilipin in these cells (Figure 4B). Gene expression of the adipogenic key regulator PPARγ2 and the adipocyte markers FABP4 and perilipin are also considerably higher in the DLK1^−^/CD34^+^/CD24^−^ ASCs (Figure 4C). Moreover, boosting of adipogenesis by the PPARγ2 agonist Troglitazone leads to an ~80 fold higher induction of the expression of PPARγ2 and to an ~300 fold higher gene expression of FABP4 and perilipin in DLK1^−^/CD34^+^/CD24^−^ ASCs than in DLK1^−^/CD34^+^/CD24^+^ cells (Supplementary Figure S1). The proliferative capacity is similar in both ASC subpopulations, as measured by BrdU incorporation and cell counting (Figure 4D,E). These data suggest that the stemness of human DLK1^−^/CD34^+^/CD24^+^ ASCs is higher than of DLK1^−^/CD34^+^/CD24^−^ ASCs, while the CD24^−^ cells have a considerably higher adipogenic potential.

### 3.5. Expression of Quiescence, Stemness, Proliferation, Senescence and Adipogenesis Markers in Routinely Isolated and In Vitro Cultivated Human DLK1^−^/CD34^+^ ASCs

Gene expression analysis in cultivated passage 2 DLK1^−^/CD34^+^ ASCs routinely isolated from the SVF by differential centrifugation, plating and plastic-adherence [16,51,52] (Supplementary Figure S2A) shows that the expression pattern of markers for quiescence, stemness, proliferation, senescence and adipogenesis is similar as in sorted DLK1^−^/CD34^+^ ASCs analyzed ex vivo, immediately after isolation (Figure 2). Pluripotency markers are barely detectable, the stemness markers KLF4 and c-MYC are highly expressed, C/EBPβ is highly expressed and the levels of C/EBPδ and PPARγ2 are relatively low. However, the expression levels of the CDKIs p27^Kip1^ and p57^Kip2^ are reduced in in vitro cultivated DLK1^−^/CD34^+^ ASCs concomitant with a significant expression of the key proliferation markers Ki67 and CycA2. Intriguingly, p21^Cip1^ is as high expressed as in ex vivo ASCs suggesting that mainly p21^Cip1^, as an G0/G1-phase cell cycle inhibitor, contributes to the long doubling time (~4 days) of proliferating primary human ASCs in culture (Figure 4) [7,53]. In fact, the analysis of the cell cycle profile of asynchronously proliferating ASCs reveals that ~92% of the cells are in the G0/G1-phase but only ~4.5% in S and ~3.5% in G2/M (Supplementary Figure S2B), indicating that the slow proliferating ASCs have a prolonged G0/G1 phase. This is in sharp contrast to the cell cycle profile of fast asynchronously proliferating embryonic stem cells (ESCs) and immortalized or transformed cell lines, which contain considerably less cells in G0/G1, as shown elsewhere. To corroborate that cultured ASCs actually proliferate despite a prolonged G0/G1 phase a serum arrest/release experiment was performed. As demonstrated in Supplementary Figure S2C, serum-arrested G0/G1 ASCs enter S-phase and progress throughout the cell cycle into G2/M after serum-restimulation.

### 3.6. Characterization of CD24 in Human ASCs

**Figure 5:**
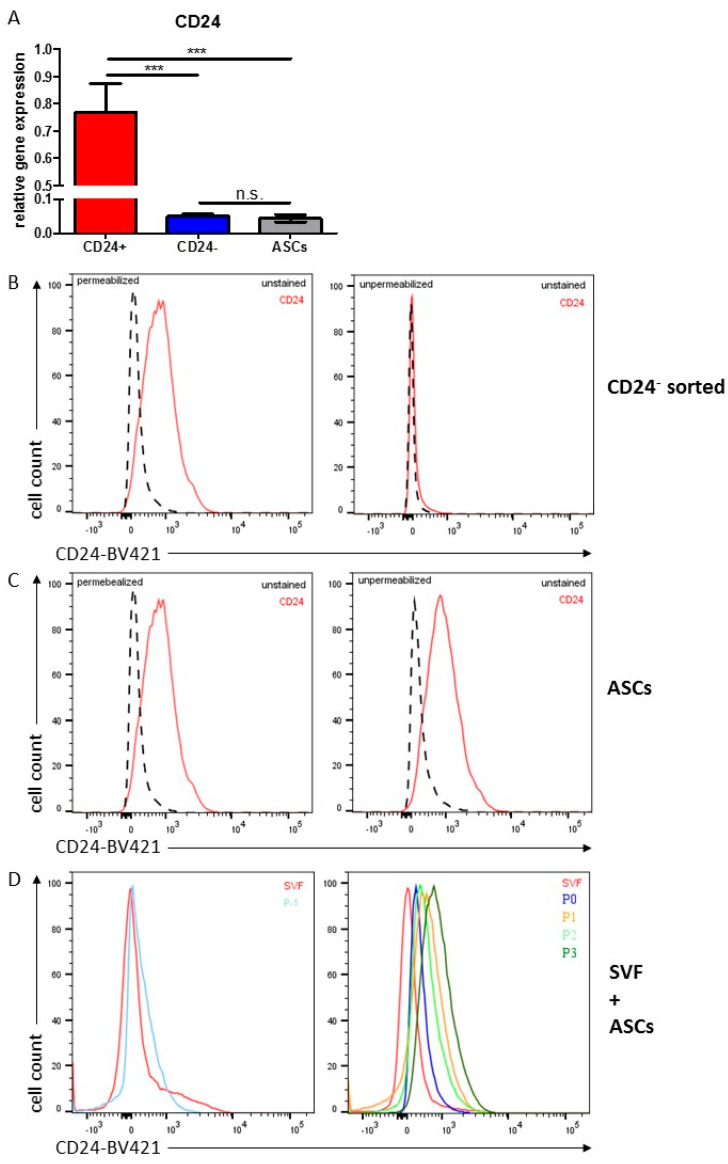
CD24 Expression in ASCs. (**A**) CD24 gene expression in sorted CD24^+^ and CD24^−^ ASCs as well as in cultivated ASCs, routinely isolated by the plastic-adherence protocol. Merged data from 4 different donors are shown, gene expression was normalized to RPS18, n = 3 for technical replicates. Mean+SEM is shown, unpaired student´s t-test was performed to calculate significances with *** *p* < 0.001. (**B**,**C**) Intracellular (left panel, permeabilized) and cell-surface (right panel, unpermeabilized) CD24 protein level measured by FACS for (**B**) sorted CD24^−^ cells and (**C**) unsorted cultivated ASCs routinely isolated by the plastic-adherence protocol at passage 2. Unstained control (black, dashed lines) and CD24 stained cells (red, solid lines) are shown. (**D**) CD24 cell-surface expression measured by FACS on ASCs in the course of routine isolation by the plastic-adherence protocol and cultivation. (Left panel) Unsorted SVF cells (red) and plastic-adherent cells after cultivation for 6 days in serum-free ASC1 medium (P-1, light blue) are shown. (Right panel) SVF cells are shown in red. P-1 cells further cultivated for 1 week in serum-containing PM4 medium were referred to as P0 and are shown in dark blue. After freezing, these cells were re-thawed, taken into culture and referred to as ASC passage 1 (P1, orange) and further cultivated to passage 2 (P2, light green) and passage 3 (P3, dark green).

Monitoring of the CD24 mRNA expression in sorted DLK1^−^/CD34^+^/CD24^+^ and DLK1^−^/CD34^+^/CD24^−^ cells showed that CD24 is expressed in both ASC types; however, CD24 is ~15 times lower expressed in DLK1^−^/CD34^+^/CD24^−^ cells (Figure 5A). Moreover, sorted cell surface DLK1^−^/CD34^+^/CD24^−^ ASCs contain intracellular CD24 protein (Figure 5B), indicating that CD24 in ASCs is regulated at the level of gene expression and subcellular localization. The analysis of the CD24 mRNA level in cultures of routinely, by plastic-adherence, isolated DLK1^−^/CD34^+^ ASCs shows that they contain similar CD24 mRNA level as ex vivo sorted DLK1^−^/CD34^+^/CD24^−^ ASCs (Figure 5A). Interestingly, monitoring of CD24 protein in passage 3 DLK1^−^/CD34^+^ ASCs shows that all cells contain intracellular as well as extracellular CD24 protein (Figure 5C). The level of CD24 protein on the surface of these cells (Figure 5C) and of sorted ex vivo DLK1^−^/CD34^+^/CD24^+^ ASCs is similar (Figure 1B). Given the low percentage of DLK1^−^/CD34^+^/CD24^+^ ASCs in the SVF and the similar proliferative activity of CD24^+^ and CD24^−^ ASCs, it is likely that routinely isolated ASC populations convert from a DLK1^−^/CD34^+^/CD24^−^ to a DLK1^−^/CD34^+^/CD24^+^ phenotype in the course of isolation or cultivation. To prove this, CD24 protein was monitored in these ASCs during their isolation by plastic-adherence and in vitro cultivation (Figure 5D). In fact, we found that these cells are initially DLK1^−^/CD34^+^/CD24^−^ and begin to express CD24 protein at the cell surface when cultivated in growth factor/mitogen containing PM4 medium, suggesting that the translocation of CD24 to the cell surface is induced by serum components.

### 3.7. Knock-Down of CD24 Reduces Proliferative and Adipogenic Capacity of Human ASCs

**Figure 6:**
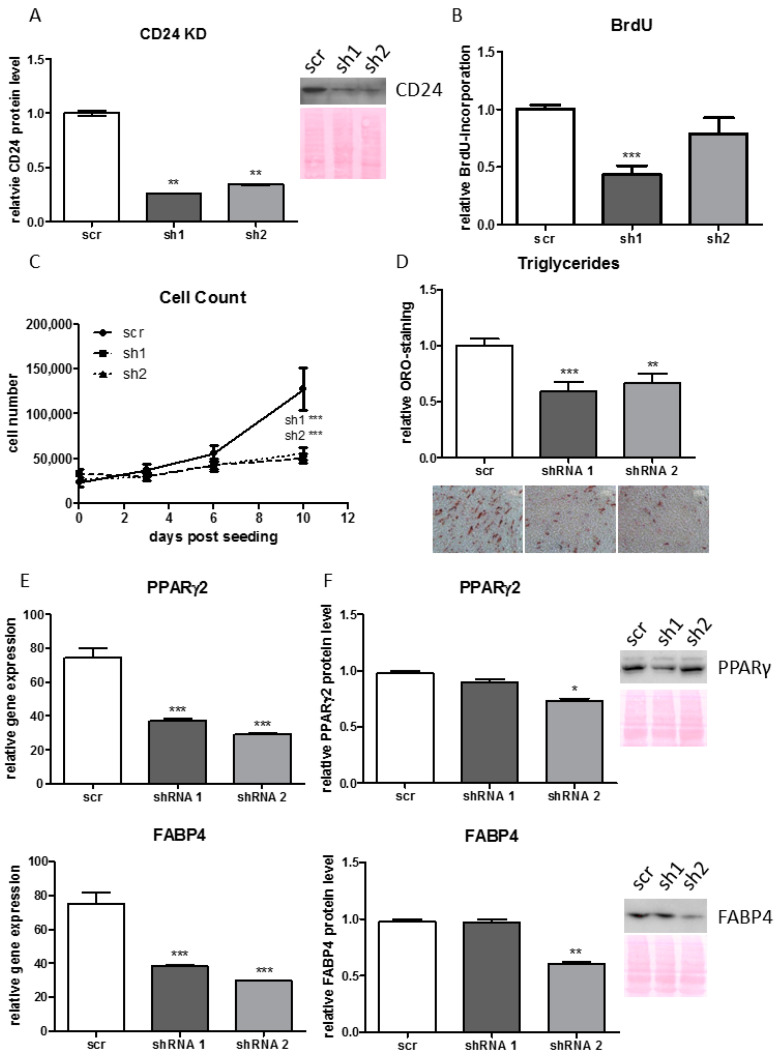
Impact of shRNA mediated CD24 Knock Down (KD) on proliferation and adipogenesis in human ASCs. (**A**) CD24 KD was performed in ASCs routinely isolated by the plastic adherence protocol and validated by Western Blot analysis. (**B**) Proliferation measured by BrdU incorporation. (**C**) Proliferation measured by cell counting. (**D**–**F**) Monitoring of adipogenic differentiation status of CD24 KD ASCs on day 14 post induction of adipogenesis. (**D**) Triglyceride accumulation measured by Oil-Red-O (ORO) staining. (**E**) Gene expression analysis for PPARγ2 and FABP4. (**F**) Western Blot analysis of PPARγ2 and FABP4 protein levels. Representative results in ASCs derived from 3 different donors are shown, n = 3 for technical replicates, gene expression was normalized to β-Actin. Mean+SEM is shown, unpaired student´s *t*-test was performed to calculate significances with * *p* < 0.05 ** *p* < 0.01 and *** *p* < 0.001.

To analyze whether CD24 is of functional importance for human ASCs it was knocked down (KD) in routinely isolated human ASCs by a lentiviral based gene-silencing approach using shRNAs (Figure 6A). Cell counting and BrdU incorporation experiments show that the CD24 KD significantly reduces the proliferation capacity of ASCs (Figure 6B,C). The adipogenic potential is also considerably decreased in CD24 KD ASCs as shown by reduced accumulation of triglycerides (Figure 6D) and reduced expression of PPARγ2 and FABP4 mRNA (Figure 6E) and protein (Figure 6F). These data indicate that CD24 is necessary for appropriate proliferation and adipogenic differentiation of human ASCs. Intriguingly, we have shown in the present study that human cell surface DLK1^−^/CD34^+^/CD24^+^ ASCs differentiate much less than DLK1^−^/CD34^+^/CD24^−^ cells (Figure 4A–C) and, similarly, Rodeheffer et al (2008) showed that mouse cell surface CD24^+^ ASCs differentiate less good into adipocytes than CD24^−^ ASCs. Together these data suggest a role of intracellular CD24 protein in the regulation of adipogenesis.

## 4. Discussion

In the present study, we analyzed the status of quiescence, stemness, proliferation, senescence and terminal adipogenic differentiation capacity in populations of human ASCs defined by the cell surface markers DLK1, CD34 and CD24 ex vivo, directly after isolation from human sWAT. Furthermore, we studied the function of CD24 in human ASCs by gene-silencing experiments.

Quiescence is an important feature of adult stem cells, considered to increase stress resistance and self-renewal capacity [21,22]. The quiescence program is mediated by cell cycle regulatory proteins in response to different stimuli that regulate quiescence versus cell cycle progression and are interconnected at the restriction point in the G1-phase of the cell cycle [54]. At this checkpoint several signals flow together regulating the activity of the retinoblastoma protein and other members of the pocket protein family, that are controlled through phosphorylation by cyclin-dependent kinase 4 (CDK4), CDK6 and CDK2, which are activated in response to mitogenic stimuli by binding to D- and E-cyclins leading to cell cycle progression into S-phase. The proliferation stimulating effects of these CDK/cyclin complexes are counteracted by CDKIs of the CIP/KIP family (p21^CIP1^, p27^KIP1^ and p57^KIP2^) that block CDK activity leading to cell cycle exit and entry into quiescence. Previous studies investigated the expression of quiescence markers in cultivated ASCs [55]. However, to our knowledge, the status of quiescence has so far not been analyzed in ex vivo ASCs. In our present study, we show considerable expression of all three quiescence markers p21^CIP1^, p27^KIP1^ and p57^KIP2^ in ASCs analyzed immediately after isolation from sWAT of several donors. Quiescence-inducing signals and the response to them can be in part different. It was shown that low transient DNA damage, induced by mild DNA replication stress in normal proliferating cells induces quiescence by the induction of p21^CIP1^ as an endogenous DNA damage response [54]. We find that the p21^Cip1^ levels are the highest among the CDKIs in quiescent ASCs and previous studies of our laboratory have detected significant DNA damage in ASCs freshly isolated from human sWAT [7,53]. Thus, it is conceivable that DNA damage contributes to the induction of p21^CIP1^ and, as a consequence, quiescence in ASCs. There is precedence that paucity of growth factors and mitogens and/or contact inhibition leads to upregulation of p27^Kip1^ and p57^KIP2^ and promotion of the quiescent state [26,54]. Such conditions frequently exist in solid tissues and paucity of growth factors and mitogens in sWAT is indicated by our finding that ASCs ex vivo do neither express G1-phase cyclins (data not shown) nor S-phase cyclins. Thus, it is very likely that paucity of growth factors and mitogens and, as a consequence, low cyclin levels and induction of p27^Kip1^ and p57^KIP2^ contribute to quiescence in ASCs of sWAT. Our findings that cyclins are down and all CIP/KIP family CDKIs (p21^CIP1^, p27^KIP1^ and p57^KIP2^) are upregulated in ASCs is in keeping with the current model that quiescence decisions rely on the expression of not only one but several CDKIs and modulation of additional proliferation-regulating signals that are integrated by their combined effects on G1- and G1/S-CDKs [54].

In routinely isolated proliferating ASCs in cell culture, we detected similar high expression levels of p21^Cip1^ as in ex vivo ASCs, while p27^Kip1^ and p57^Kip2^ were considerably lower expressed, likely due to the high growth factor/mitogen levels in FBS containing cell culture medium. Given that early passage ASCs contain moderate levels of DNA damage and proliferate slow [7,53] and that the majority of the ASC population (>90%) are most of the time in the G0/G1 phase of the cell cycle [55], it is likely that mild DNA damage stress occurring during normal cell division in cultivated ASCs leads to moderate induction of p21^Cip1^ and decelerated progression through the G0/G1-phase of the cell cycle. Since we more recently showed that ~80% of ASCs at passage 5 robustly express Ki67 [7], one can conclude that the majority of ASCs in asynchronous culture are in the G1-phase of the cell cycle while some ASCs are in G0.

While according to the current model low, transient DNA-damage is the initial stimulus triggering a moderate p53/p21^CIP1^ response leading to cell cycle arrest and exit to quiescence [54], sustained and robust activation of the DNA-damage response p53/p21^CIP1^ pathway promotes induction of the p16^Ink4a^/RB cell cycle inhibitory pathway, further accumulation of the CDKIs p21^CIP1^ and p16^Ink4a^ and eventually cellular senescence [56,57]. We could barely detect p16^Ink4A^ expression in ASCs isolated from 5 out of 6 donors while ASCs from 1 out of 6 donors expressed significant but still low amounts of p16^Ink4A^ mRNA. This is in accordance with a more recent study of our laboratory demonstrating that only ~5% of routinely cultivated ASCs in passage 5 express senescence markers [7,53]. These findings indicate that the vast majority of ASCs in sWAT are in a quiescent but not senescent state. However, our data underscore also that senescent ASCs exist in sWAT, as has been previously shown especially in conditions of obesity, diabetes and aging [6,7,9,58,59].

Since contradictory results exist about the expression of NANOG, SOX2 and OCT4 in adult stem cells [29,30,31,32,33,34,35,60], it was interesting to analyze the expression of these pluripotency factors in ASCs ex vivo immediately after their isolation from sWAT. Our study shows that all 3 factors are hardly detectable in ASCs from 5 out of 6 donors while ASCs isolated from one donor showed low but significant expression of the three pluripotency factors with OCT4 mRNA levels being the highest. This indicates that in the majority of ASCs NANOG, OCT4 and SOX2 are hardly detectable; however, rare conditions may exist, as for example shown in the environment of senescent cells [61], in which pluripotency factors are significantly expressed in human ASCs. In our study, a high expression of the CDK inhibitors p21^CIP1^, p27^KIP1^ and p57^KIP2^ and a non-detectability of the CDK activators cyclin D1 - D3 correlate with the absence of NANOG, OCT4 and SOX2 in ASCs. It is interesting to note, that recent findings indicate that low levels of G1-cyclins and the inhibition of CDK activity leads to proteasomal degradation and, hence, clearance of pluripotency factors in ESCs [62]. The question whether similar as in ESCs low CDK activity and the proteasome-ubiquitin system contributes to the absence of NANOG, OCT4 and SOX2 in ASCs warrants further studies.

Cell cycle arrest by quiescence-inducing signals suppresses terminal differentiation in human diploid fibroblasts [63] suggesting that unique signaling mechanisms are linked to the quiescence state to promote the action of multipotency factors and/or to inhibit terminal differentiation transcriptional programs. In our study, we show that the somatic cell reprogramming factors, c-MYC and KLF4 [36], which have been shown to ensure high stemness in hematopoietic stem cells by balancing quiescence, proliferation and differentiation [38,39], are strongly expressed ex vivo in ASCs isolated from 6 out of 6 donors. Interestingly, previous studies suggest that both c-MYC and KLF4 act at a hub at which growth arrested murine 3T3-L1 preadipocytes are either directed toward cell cycle re-entry or committed to terminal differentiation [45,46,47]. Moreover, KLF4 has been shown to activate expression of p21^CIP1^, thereby suppressing cell proliferation [42]. This anti-proliferative function of KLF4 can be counteracted by c-MYC, which suppresses the expression of p21^CIP1^ in response to DNA damage [40]. Thus, our data that c-MYC, KLF4 and p21^CIP1^ are highly expressed in quiescent ASCs warrants further studies to analyze whether the balance between these factors is important for the maintenance of the quiescent state and hence stemness in ASCs.

KLF4 acts as an inducer of the early adipogenic transcription factor C/EBPβ in 3T3-L1 preadipocytes [46]. It has been shown that C/EBPβ is already expressed in murine 3T3-L1 preadipocytes [64,65] and human ASCs [66] before adipogenic stimulation and it is particularly important that C/EBPβ is genome-wide bound to almost all quiescent adipogenic regions of open chromatin, where it is required for the formation of adipogenic transcription factor complexes to induce the adipogenic differentiation program [48]. In our study, we show that KLF4 and C/EBPβ but not C/EBPδ and PPARγ2, are always highly expressed in quiescent ASCs. This suggests a scenario, in which the KLF4/C/EBPβ axis is already established in quiescent ASCs in sWAT and C/EBPβ serves as origin for the formation of PPARγ2-containing adipogenic transcription factor complexes that induce adipogenic differentiation after given stimuli.

CD24 is expressed by various stem cell types and plays a role in the regulation of cell proliferation and differentiation [24]. The comparison of ex vivo DLK1^−^/CD34^+^/CD24^−^ and DLK1^−^/CD34^+^/CD24^+^ ASC populations from several donors in our study demonstrated that KLF4 and c-MYC are higher expressed in the DLK1^−^/CD34^+^/CD24^+^ population correlating with a higher colony formation capacity and a considerably lower adipogenic differentiation activity. Thus, the balance between c-MYC and KLF4 may be important for stem-like capacity (stemness) and adipogenic differentiation potential of human CD24^+^ vs CD24^−^ ASCs and stemness is most likely higher in CD24^+^ ASCs than in CD24^−^ ASCs in human sWAT. Similar data were shown in the mouse model. CD24 was identified as a marker for high stem-like capacity of murine ASCs; while both CD24^+^ and CD24^−^ subpopulations can undergo proliferation and adipogenic differentiation in cell culture only CD24^+^ ASCs are capable of reconstituting functional WAT depots in A-Zip lipodystrophic mice [2]. Moreover, data suggest that CD24^+^ cells give rise to CD24^−^ cells in mice during adipogenesis [67]. In the present study, we found no differences regarding the expression levels of the quiescence markers p21^Cip1^, p27^Kip1^ and p57^Kip2^, the proliferation markers Ki67 and Cyclin A2 and the senescence marker p16^Ink4a^ between CD24^−^ and CD24^+^ ASCs and the pluripotency markers are hardly detectable in both ASC types. Thus, in human sWAT both CD24 ASC types are in the quiescent state and show almost no pluripotent capacity. Analyzing the role of CD24 in human ASCs routinely isolated from the SVF and propagated in cell culture revealed that ex vivo DLK1^−^/CD34^+^/CD24^−^ ASCs most likely convert to DLK1^−^/CD34^+^/CD24^+^ ASCs in the course of cultivation in PM4 cell culture medium containing growth factors and mitogens. Intriguingly, silencing of CD24 in these cells reduces both proliferation and adipogenic differentiation capacity. This suggests that CD24 is necessary for adequate ASC proliferation and adipogenesis although ex vivo cell-surface DLK1^−^/CD34^+^/CD24^−^ ASCs differentiate much stronger into adipocytes than DLK1^−^/CD34^+^/CD24^+^ ASCs. Based on these data one could hypothesize that intracellular CD24 protein plays a role in the regulation of ASC proliferation and adipogenesis. This hypothesis is supported by a study showing that CD24 is not only localized at the cell surface but also in the nucleus [68]. The same study shows that intracellular CD24 protein plays a role in the regulation of proliferation and differentiation. The complete elucidation of the mechanisms underlying the effects of CD24 in human ASCs warrants further studies.

Beside the importance of our new findings for the basic understanding of the biology of human ASCs, the identification of DLK1^−^/CD34^+^/CD24^+^ ASCs as stromal cells with high stemness also underscores their importance as multipotent stromal cells for applications in regenerative medicine and tissue engineering, as reviewed elsewhere [12,13,14,69].

## 5. Conclusions

In conclusion, we show that the vast majority of ASCs in human sWAT exist in a quiescent state, express high levels of the somatic stemness factors c-MYC and KLF4 and of the early adipogenic transcription factor C/EBP β but barely senescence and pluripotency markers. Moreover, our data indicate that stemness is higher and adipogenic capacity much lower in DLK1^−^/CD34^+^/CD24^+^ relative to DLK1^−^/CD34^+^/CD24^−^ ASC subpopulations and that CD24 is necessary for adequate ASC proliferation and adipogenic differentiation. Furthermore, the present study demonstrates that human ASCs, which are routinely isolated from the SVF by differential centrifugation, plating and plastic-adherence and further cultivated in vitro, express the functional cell-surface marker combination DLK1^−^/CD34^+^/CD24^+^ and possess high stemness and relatively low adipogenic differentiation capacity.

## Data Availability

The data supporting the findings of this study are included within the article.

## Supplementary Materials

The following data are available online at https://www.mdpi.com/2073-4409/10/2/214/s1. Table S1: Clinical anthropometric parameters of the donors; Table S2: Primer sequences; Figure S1: Adipogenic differentiation boosted with Troglitazone; Figure S2: Marker gene expression and cell cycle profile in routinely cultivated ASCs.

## References

[1] Spalding K.L., Arner E., Westermark P.O., Bernard S., Buchholz B.A., Bergmann O., Blomqvist L., Hoffstedt J., Näslund E., Hassan M. (2008). Dynamics of fat cell turnover in humans. Nature.

[2] Rodeheffer M.S., Birsoy K., Friedman J.M. (2008). Identification of white adipocyte progenitor cells in vivo. Cell.

[3] Yamamoto N., Akamatsu H., Hasegawa S., Yamada T., Nakata S., Ohkuma M., Miyachi E., Marunouchi T., Matsunaga K. (2007). Isolation of multipotent stem cells from mouse adipose tissue. J. Derm. Sci..

[4] Tang W., Zeve D., Suh J.M., Bosnakovski D., Kyba M., Hammer R.E., Tallquist M.D., Graff J.M. (2008). White fat progenitor cells reside in the adipose vasculature. Science.

[5] Vishvanath L., Gupta R.K. (2019). Contribution of adipogenesis to healthy adipose tissue expansion in obesity. J. Clin. Investig..

[6] Minamino T., Dai Z., Jabbari A., Cerise J.E., Higgins C.A., Gong W., de Jong A., Harel S., DeStefano G.M., Rothman L. (2009). A crucial role for adipose tissue p53 in the regulation of insulin resistance. Nat. Med..

[7] Mitterberger M.C., Lechner S., Mattesich M., Zwerschke W. (2014). Adipogenic differentiation is impaired in replicative senescent human subcutaneous adipose-derived stromal/progenitor cells. J. Gerontol. Ser. A Biol. Sci. Med. Sci..

[8] Berry D.C., Jiang Y., Arpke R.W., Berglund E.D., Kyba M., Graff J.M. (2017). Cellular aging contributes to failure of cold-induced beige adipocyte formation in old mice and humans. Cell Metab..

[9] Guillermier C., Fazeli P.K., Kim S., Lun M., Zuflacht J.P., Milian J., Lee H., Francois-Saint-Cyr H., Horreard F., Larson D. (2017). Imaging mass spectrometry demonstrates age-related decline in human adipose plasticity. JCI Insight.

[10] Ghaben L., Scherer P.E. (2019). Adipogenesis and metabolic health. Nat. Rev. Mol. Cell Biol..

[11] Gimble J.M., Bunnell B.A., Chiu E.S., Guilak F. (2011). Concise review: Adipose-derived stromal vascular fraction cells and stem cells: Let’s not get lost in translation. Stem. Cells.

[12] Ballini S., Scacco S., Coletti D., Pluchino S., Tatullo M. (2017). Mesenchymal stem cells as promoters, enhancers, and playmakers of the translational regenerative medicine. Stem. Cells Int..

[13] Ballini S., Cantore S., Scacco S., Coletti D., Tatullo M. (2018). Mesenchymal stem cells as promoters, enhancers, and playmakers of the translational regenerative medicine 2018. Stem. Cells Int..

[14] Spagnuolo G., Codispoti B., Marrelli M., Rengo C., Rengo S., Tatullo M. (2018). Commitment of oral-derived stem cells in dental and maxillofacial applications. Dent. J..

[15] Zimmerlin L., Donnenberg V.S., Pfeifer M.E., Meyer E.M., Péault B., Rubin J.P., Donnenberg A.D. (2010). Stromal vascular progenitors in adult human adipose tissue. Cytometry. Part A J. Int. Soc. Analyt. Cytol..

[16] Zwierzina M.E., Ejaz A., Bitsche M., Blumer M.J.F., Mitterberger M.C., Mattesich M., Amann A., Kaiser A., Pechriggl E.J., Hörl S. (2015). Characterization of DLK1(PREF1) + /CD34+ cells in vascular stroma of human white adipose tissue. Stem. Cell Res..

[17] Frazier T.P., Bowles A., Lee S., Abbott R., Tucker H.A., Kaplan D., Wang M., Strong A., Brown Q., He J. (2016). Serially transplanted nonpericytic CD146(-) adipose stromal/stem cells in silk bioscaffolds regenerate adipose tissue in vivo. Stem. Cells.

[18] Hudak S., Sul H.S. (2013). Pref-1, a gatekeeper of adipogenesis. Front. Endocrinol (Lausanne).

[19] Traustadottir G.A., Kosmina R., Sheikh S.P., Jensen C.H., Andersen D.C. (2013). Preadipocytes proliferate and differentiate under the guidance of Delta-like 1 homolog (DLK1). Adipocyte.

[20] Sengenès C., Lolmède K., Zakaroff-Girard A., Busse R., Bouloumié A. (2005). Preadipocytes in the human subcutaneous adipose tissue display distinct features from the adult mesenchymal and hematopoietic stem cells. J. Cell Physiol..

[21] Sidney L.E., Branch M.J., Dunphy S.E., Dua H.S., Hopkinson A. (2014). Concise review: Evidence for CD34 as a common marker for diverse progenitors. Stem. Cells.

[22] Sammar M., Gulbins E., Hilbert K., Lang F., Altevogt P. (1997). Mouse CD24 as a signaling molecule for integrin-mediated cell binding: Functional and physical association with src-kinases. Biochem. Biophys. Res. Commun..

[23] Fang X., Zheng P., Tang J., Liu Y. (2010). CD24: From A to Z. Cell Mol. Immunol..

[24] Shackleton M., Vaillant F., Simpson K.J., Stingl J., Smyth G.K., Asselin-Labat M., Wu L., Lindeman G.J., Visvader J.E. (2006). Generation of a functional mammary gland from a single stem cell. Nature.

[25] Shakiba N., White C.A., Lipsitz Y.Y., Yachie-Kinoshita A., Tonge P.D., Hussein S.M.I., Puri M.C., Elbaz J., Morrissey-Scoot J., Li M. (2015). CD24 tracks divergent pluripotent states in mouse and human cells. Nat. Commun..

[26] Tesio M., Trumpp A. (2011). Breaking the cell cycle of HSCs by p57 and friends. Cell. Stem. Cell.

[27] van Velthoven T.J., Rando T.A. (2019). Stem cell quiescence: Dynamism, restraint, and cellular idling. Cell. Stem. Cell.

[28] Boyer L.A., Lee T.I., Cole M.F., Johnstone S.E., Levine S.S., Zucker J.P., Guenther M.G., Kumar R.M., Murray H.L., Jenner R.G. (2005). Core transcriptional regulatory circuitry in human embryonic stem cells. Cell.

[29] Izadpanah R., Trygg C., Patel B., Kriedt C., Dufour J., Gimble J.M., Bunnell B.A. (2006). Biologic properties of mesenchymal stem cells derived from bone marrow and adipose tissue. J. Cell Biochem..

[30] Berg J.S., Goodell M.A. (2007). An argument against a role for Oct4 in somatic stem cells. Cell. Stem Cell.

[31] Greco S.J., Liu K., Rameshwar P. (2007). Functional similarities among genes regulated by OCT4 in human mesenchymal and embryonic stem cells. Stem. Cells.

[32] Lengner C.J., Camargo F.D., Hochedlinger K., Scholer H.R., Tomilin A., Jaenisch R. (2007). Oct4 expression is not required for mouse somatic stem cell self-renewal. Cell. Stem. Cell.

[33] Liedtke S., Enczmann J., Waclawczyk S., Wernet P., Kögler G. (2007). Oct4 and its pseudogenes confuse stem cell research. Cell. Stem. Cell.

[34] Kaltz N., Funari A., Hippauf S., Delorme B., Noël D., Riminucci M., Jacobs V.R., Häupl T., Jorgensen C., Charbord P. (2008). In vivo osteoprogenitor potency of human stromal cells from different tissues does not correlate with expression of POU5F1 or its pseudogenes. Stem. Cells.

[35] Riekstina U., Cakstina I., Parfejevs V., Hoogduijn M., Jankovskis G., Muiznieks I., Muceniece R., Ancans J. (2009). Embryonic stem cell marker expression pattern in human mesenchymal stem cells derived from bone marrow, adipose tissue, heart and dermis. Stem. Cell. Rev. Rep..

[36] Takahashi K., Yamanaka S. (2006). Induction of pluripotent stem cells from mouse embryonic and adult fibroblast cultures by defined factors. Cell.

[37] Takahashi K., Tanabe K., Ohnuki M., Narita M., Ichisaka T., Tomoda K., Yamanaka S. (2007). Induction of pluripotent stem cells from adult human fibroblasts by defined factors. Cell.

[38] Laurenti E., Wilson A., Trumpp A. (2009). Myc’s other life: Stem cells and beyond. Curr. Opin. Cell. Biol..

[39] Park K.W., Halperin D.S., Tontonoz P. (2008). Before they were fat: Adipocyte progenitors. Cell Metab..

[40] Seoane J., Le H.V., Massagué J. (2002). Myc suppression of the p21(Cip1) Cdk inhibitor influences the outcome of the p53 response to DNA damage. Nature.

[41] Shields J.M., Christy R.J., Yang V.W. (1996). Identification and characterization of a gene encoding a gut-enriched Krüppel-like factor expressed during growth arrest. J. Biol. Chem..

[42] Zhang W., Geiman D.E., Shields J.M., Dang D.T., Mahatan C.S., Kaestner K.H., Biggs J.R., Kraft A.S., Yang V.W. (2000). The gut-enriched Kruppel-like factor (Kruppel-like factor 4) mediates the transactivating effect of p53 on the p21WAF1/Cip1 promoter. J. Biol. Chem..

[43] Yusuf I., Kharas M.G., Chen J., Peralta R.Q., Maruniak A., Sareen P., Yang V.W., Kaestner K.H., Fruman D.A. (2008). KLF4 is a FOXO target gene that suppresses B cell proliferation. Int. Immunol..

[44] McConnell B.B., Yang V.W. (2010). Mammalian Krüppel-like factors in health and diseases. Physiol. Rev..

[45] Pulverer B., Sommer A., McArthur G.A., Eisenman R.N., Lüscher B. (2000). Analysis of Myc/Max/Mad network members in adipogenesis: Inhibition of the proliferative burst and differentiation by ectopically expressed Mad1. J. Cell. Physiol..

[46] Birsoy Z., Chen E.C., Friedman J. (2008). Transcriptional regulation of adipogenesis by KLF4. Cell Metab..

[47] Ntambi M., Young-Cheul K. (2000). Adipocyte differentiation and gene expression. J. Nutr..

[48] Lefterova I., Haakonsson A.K., Lazar M.A., Mandrup S. (2014). PPARγ and the global map of adipogenesis and beyond. Trends Endocrinol. Metab..

[49] Cristancho G., Lazar M.A. (2011). Forming functional fat: A growing understanding of adipocyte differentiation. Nat. Rev. Mol. Cell Biol..

[50] Mandl M., Wagner S.A., Hatzmann F.M., Mitterberger-Vogt M.C., Zwierzina M.E., Mattesich M., Zwerschke W. (2019). Sprouty1 is a weight-loss target gene in human adipose stem/progenitor cells that is mandatory for the initiation of adipogenesis. Cell. Death Dis..

[51] Mitterberger C., Lechner S., Mattesich M., Kaiser A., Probst D., Wenger N., Pierer G., Zwerschke W. (2012). DLK1(PREF1) is a negative regulator of adipogenesis in CD105(+)/CD90(+)/CD34(+)/CD31(-)/FABP4(-) adipose-derived stromal cells from subcutaneous abdominal fat pats of adult women. Stem. Cell Res..

[52] Mitchell J.B., McIntosh K., Zvonic S., Garrett S., Floyd Z.E., Kloster A., di Halvorsen Y., Storms R.W., Goh B., Kilroy G. (2006). Immunophenotype of human adipose-derived cells: Temporal changes in stromal-associated and stem cell-associated markers. Stem. Cells.

[53] Mitterberger M.C., Mattesich M., Zwerschke W. (2014). Bariatric surgery and diet-induced long-term caloric restriction protect subcutaneous adipose-derived stromal/progenitor cells and prolong their life span in formerly obese humans. Exp. Gerontol..

[54] Pack L.R., Daigh L.H., Meyer T. (2019). Putting the brakes on the cell cycle: Mechanisms of cellular growth arrest. Curr. Opin. Cell. Biol..

[55] Søndergaard R.H., Follin B., Lund L.D., Juhl M., Ekblond A., Kastrup J., Haack-Sørensen M. (2017). Senescence and quiescence in adipose-derived stromal cells: Effects of human platelet lysate, fetal bovine serum and hypoxia. Cytotherapy.

[56] van Deursen J.M. (2014). The role of senescent cells in ageing. Nature.

[57] Salotti J., Johnson P.F. (2019). Regulation of senescence and the SASP by the transcription factor C/EBPβ. Exp. Gerontol..

[58] Baker D.J., Childs B.G., Durik M., Wijers M.E., Sieben C.J., Zhong J., Saltness R.A., Jeganathan K.B., Verzosa G.C., Pezeshki A. (2016). Naturally occurring p16(Ink4a)-positive cells shorten healthy lifespan. Nature.

[59] Xu M., Palmer A.K., Ding H., Weivoda M.M., Pirtskhalava T., White T.A., Sepe A., Johnson K.O., Stout M.B., Giorgadze N. (2015). Targeting senescent cells enhances adipogenesis and metabolic function in old age. Elife.

[60] Pierantozzi E., Gava B., Manini I., Roviello F., Marotta G., Chiavarelli M., Sorrentino V. (2011). Pluripotency regulators in human mesenchymal stem cells: Expression of NANOG but not of OCT-4 and SOX-2. Stem. Cells Dev..

[61] Chiche A., Chen C., Li H. (2020). The crosstalk between cellular reprogramming and senescence in aging and regeneration. Exp. Gerontol..

[62] Liu L., Michowski W., Kolodziejczyk A., Sicinski P. (2019). The cell cycle in stem cell proliferation, pluripotency and differentiation. Nat. Cell Biol..

[63] Coller A., Sang L., Roberts J.M. (2006). A new description of cellular quiescence. PLoS Biol..

[64] Lefterova M.I., Zhang Y., Steger D.J., Schupp M., Schug J., Cristancho A., Feng D., Zhuo D., Stoeckert C.J., Liu X.S. (2008). PPARgamma and C/EBP factors orchestrate adipocyte biology via adjacent binding on a genome-wide scale. Genes Dev..

[65] Siersbæk R., Nielsen R., John S., Sung M.-H., Baek S., Loft A., Hager G.L., Mandrup S. (2011). Extensive chromatin remodelling and establishment of transcription factor ‘hotspots’ during early adipogenesis. EMBO J..

[66] Mikkelsen T.S., Xu Z., Zhang X., Gimble J.M., Lander E.S., Rosen E.D. (2010). Comparative epigenomic analysis of murine and human adipogenesis. Cell.

[67] Berry R., Rodeheffer M.S. (2013). Characterization of the adipocyte cellular lineage in vivo. Nat. Cell. Biol..

[68] Wang L., Liu R., Ye P., Wong C., Chen G., Zhou P., Sakabe K., Zheng X., Wu W., Zhang P. (2015). Intracellular CD24 disrupts the ARF-NPM interaction and enables mutational and viral oncogene-mediated p53 inactivation. Nat. Commun..

[69] Marrelli M., Pujia A., Palmieri F., Gatto R., Falisi G., Gargari M., Caruso S., Apicella D., Rastelli C., Nardi G.M. (2016). Innovative approach for the in vitro research on biomedical scaffolds designed and customized with CAD-CAM technology. Int. J. Immunopathol. Pharmacol..

