# Supplementary material for: Quiescence, Stemness and Adipogenic Differentiation Capacity in Human DLK1−/CD34+/CD24+ Adipose Stem/Progenitor Cells
Source: Cells. 2021 Jan 22;10(2):214. doi: 10.3390/cells10020214 (PMC7912596; doi:10.3390/cells10020214)
Supplement: Supplementary file 1 [file cells-10-00214-s001.pdf]

Supplementary Table S1. Clinical anthropometric parameters of the donors.

Donor	Sex	Age (Years)	Height (cm)	Weight (kg)	BMI
1	f	47	150	59	26.22
2	f	57	174	84	27.74
3	f	33	160	60	23.44
4	f	38	160	60	23.44
5	f	49	161	64	24.69
6	m	25	180	125	38.58
7	f	34	165	68	24.98
8	f	23	175	74	24.16
9	f	33	153	53	22.64
10	m	31	193	115	30.87
11	f	29	152	59	25.54
12	f	37	162	50	19.05
13	m	49	182	105	31.7
14	f	35	167	62	22.23
15	f	29	172	68	22.99
16	m	18	183	82	24.49

Supplementary Table 2. Primer sequences.

Name	Sequence 5′–3′
Sox2 forward	GGGAAATGGGAGGGGTGCAAAAGAGG
Sox2 reverse	TTGCGTGAGTGTGGATGGGATTGGTG
Oct4 forward	GACAGGGGGAGGGGAGGAGCTAGG
Oct4 reverse	CTTCCCTCCAACCAGTTGCCCCAAAC
cMyc forward	GCGTCCTGGGAAGGGAGATCCGGAGC
cMyc reverse	TTGAGGGGCATCGTCGCGGGAGGCTG
NANOG forward	CAGCCCCGATTCTTCCACCAGTCCC
NANOG reverse	CGGAAGATTCCCAGTCGGGTTCACC
KLF4 forward	GAAATTCGCCCGCTCCGATGA
KLF4 reverse	CTGTGTGTTTGCGGTAGTGCC
Ki67 forward	TGACCCTGATGAGAAAGCTCAA
Ki67 reverse	CCCTGAGCAACACTGTCTTTT
p16 forward	CCCCCACTACCGTAAATCTCCAT
p16 reverse	CTGCCATTTGCTAGCAGTGTGACT
p21 forward	AGACCAGCATGACAGATTTC
p21 reverse	ACTGAGACTAAGGCAGAAGA
p27 forward	CGGGGTATGAAGAGCTTGCTTTGAT
p27 reverse	AACATTCAAAACTCCCAAGCACCTC
p57 forward	GATCAAGAAGCTGTCCGGGC
p57 reverse	TTGCTGCTACATGAACGGTCC
CyclinA2 forward	TTGATAGATGCTGACCCATACCTC
CyclinA2 reverse	ATGATTCAGGCCAGCTTTGTCC
C/EBPβ forward	AAG CAC AGC GAC GAG TAC AA
C/EBPβ reverse	AGC TGC TCC ACC TTC TTC TG
C/EBPδ forward	TCAGCAACGACCCATACCTCAG
C/EBPδ reverse	CTTTGCGCTCCTATGTCCCAAG
PPARg2 forward	ATG GGT GAA ACT CTG GGA GA
PPARg2 reverse	TGG AAT GTC TTC GTA ATG TGG A

FABP4 forward	TACTGGGCCAGGAATTTGAC
FABP4 reverse	GTGGAAGTGACGCCTTTCAT
Perilipin forward	GACAACGTGGTGGACACAGT
Perilipin reverse	CTGGTGGGTTGTCGATGTC
RPS18 forward	GGCCGAAGATATGCTCATGTGG
RPS18 reverse	TGGTGATCACACGTTCCACCTC

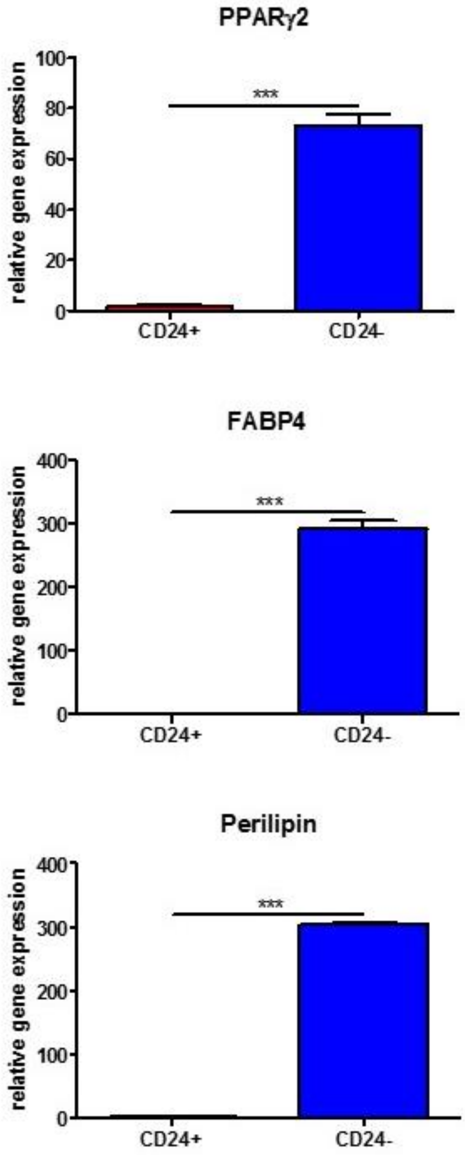

Supplementary Figure S1. Adipogenic differentiation boosted with Troglitazone**.** Adipogenic differentiation was induced by hormone cocktail plus 5 μM Troglitazone and measured after 9 days by RT-qPCR for PPARγ2, FABP4 and Perilipin. n = 3, gene expression was normalized to RPS18, unpaired student´s t-test was performed to calculate significances with *** $p < 0.001$.

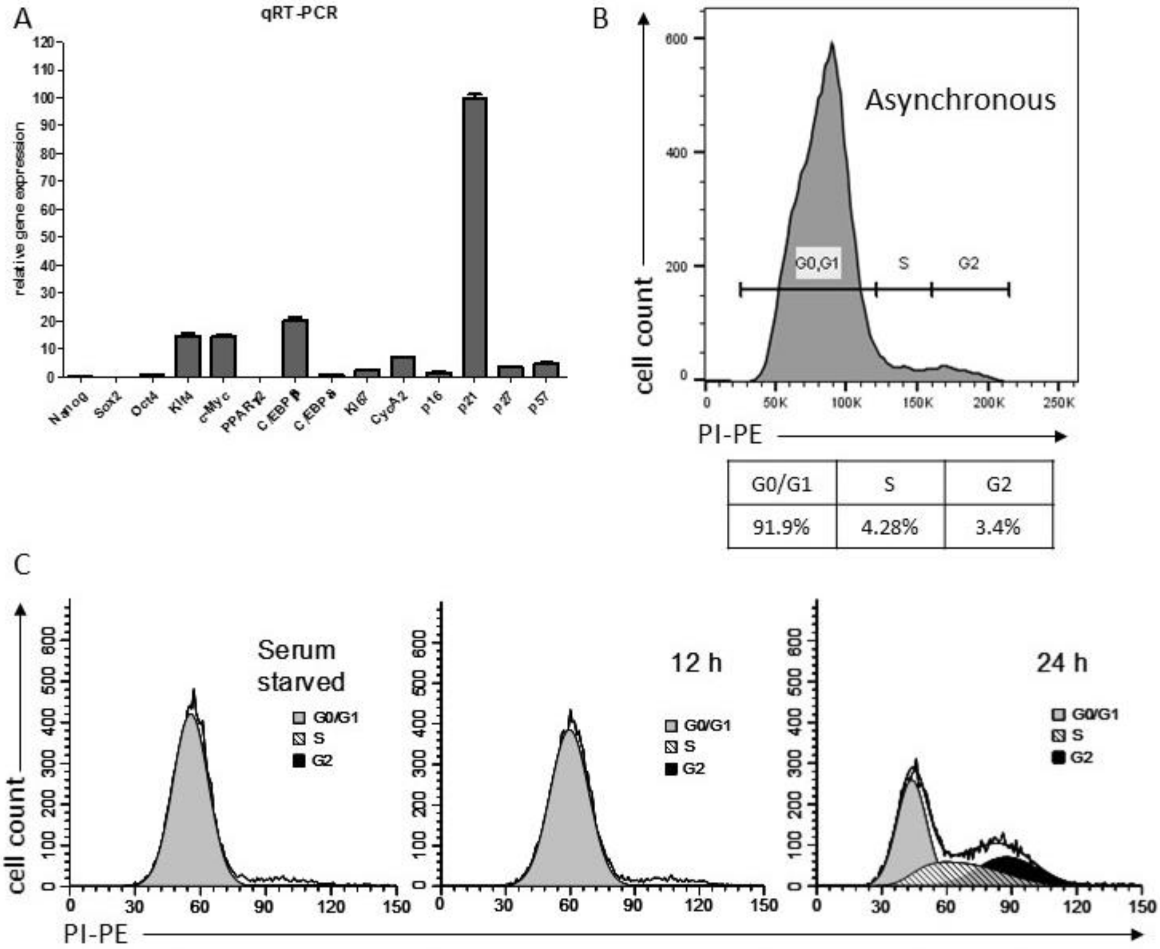

G0/G1	S	G2
91.9%	4.28%	3.4%

G0/G1	S	G2
100%	-	-

G0/G1	S	G2
100%	-	-

G0/G1	S	G2
49.8%	23.4%	26.8%

Supplementary Figure S2. Marker gene expression and cell cycle profile in routinely cultivated ASCs. ASCs routinely isolated by the plastic adherence protocol and stored at −80 °C were taken into culture and grown until passage 2 (14 days in culture). ASCs were harvested at 70% confluence and lyzed for RNA isolation to perform RT-qPCR for gene expression analysis of Cyclin A2, Ki67 (proliferation), $p16^{INK4a}$ (senescence), $p21^{Cip1}$, $p27^{Kip1}$, $p57^{Kip2}$ (quiescence), NANOG, OCT4, SOX2 (pluripotency), KLF4, c-MYC, (stemness), PPARγ2, C/EBPβ and C/EBPδ (adipogenesis). Mean + SEM is shown, n = 4 donors, gene expression normalized to RPS18. (**B**) Cell cycle profile of normal asynchronously proliferating human ASCs. To measure this, the DNA of the cells was stained by propidium iodide and analyzed by FACS. (**C**) Cell cycle profile of ASCs in a serum-arrest/release experiment. ASCs were serum starved for 2 days and the cell cycle profile was monitored before and after re-addition of serum at indicated time points.
